# Left atrial rupture during on-pump beating coronary artery bypass grafting

**DOI:** 10.1186/s40792-024-02067-6

**Published:** 2024-11-21

**Authors:** Hideaki Hidaka, Tatsuaki Sadanaga, Takafumi Hirota, Tatsuya Horibe, Jun Takaki, Kosaku Nishigawa, Takashi Yoshinaga, Toshihiro Fukui

**Affiliations:** Department of Cardiovascular Surgery, Kumamoto University Hospital, Kumamoto, Japan

**Keywords:** Left atrial rupture, On-pump beating coronary artery bypass grafting, Mitral regurgitation

## Abstract

**Background:**

On-pump beating coronary artery bypass grafting (CABG) is a procedure that uses cardiopulmonary bypass to maintain circulation and it is a useful technique for CABG in patients with severely impaired cardiac function. Here, we report a case of left atrial rupture that occurred during CABG. Reports of left atrial injury are rare, and there have been no previous reports of such cases associated with on-pump beating CABG.

**Case presentation:**

An 80-year-old man with a history of myocardial infarction was admitted to another hospital for acute heart failure. Coronary angiography revealed triple-vessel disease, and echocardiography showed reduced left ventricular function and moderate mitral regurgitation. He was transferred to our hospital for coronary artery bypass grafting and the operation was scheduled. Surgery was started with the intention of off-pump CABG, but due to circulatory instability, the patient was converted to on-pump beating CABG. While the heart was being dislocated and anastomosis was being performed, sudden bleeding from the left atrium occurred. To achieve hemostasis, we needed to arrest the patient's heart. A 5-cm laceration along the posterior mitral annulus was found in the left atrium and repaired with a bovine pericardial patch. Mitral annuloplasty with a flexible ring was performed simultaneously. He recovered uneventfully.

**Conclusions:**

The left atrial rupture during on-pump beating coronary artery bypass grafting is extremely rare. The wall of the atrium is thought to have been damaged by the stress applied during the displacement of the heart and the impact of the enlarged mitral regurgitant jet. Repair under cardiac arrest is necessary, and in some cases, mitral annuloplasty may be additionally required.

## Background

An on-pump beating CABG is a hybrid procedure that combines the potential benefits of off-pump CABG and conventional on-pump CABG. It maintains hemodynamic stability by cardiopulmonary bypass and facilitates revascularization of posterior territories. This procedure reduces myocardial ischemia and preserves cardiac function without inducing cardioplegic arrest. On-pump beating CABG is an effective substitute for off-pump CABG (OPCAB) in hemodynamically high-risk populations.

Herein, we report a rare case of left atrial rupture during on-pump beating CABG that was successfully repaired using a bovine pericardial patch.

## Case presentation

An 80-year-old man with acute heart failure was admitted to another hospital. He had previously undergone percutaneous coronary intervention but had self-discontinued medications including aspirin. After oxygenation improved with diuretics, coronary angiography revealed triple-vessel disease. Transthoracic echocardiography showed severe left ventricular dilation (diastolic dimension 60.8 mm, systolic dimension 57.2 mm) with an ejection fraction of 21.6%. Mild-to-moderate mitral regurgitation and a left atrial volume index of 45.8 mL/m^2^ were noted. He was recommended to undergo CABG and was transferred to our hospital. We planned isolated CABG and Mitral regurgitation was mild on reexamination, mitral valvuloplasty was omitted.

After median sternotomy, bilateral internal thoracic arteries and the saphenous vein Graft (SVG) from the left lower leg were harvested. Heparin (300 IU/kg) was administered after opening the pericardial cavity. An Acrobat heart positioner (Getinge, Gothenburg, Sweden) and an OPHIDIA stabilizer (VITAL, Tokyo, Japan) were used for coronary artery exposure. In order to anastomose to the obtuse marginal branch (OM), the right internal thoracic artery (RITA) was passed through the transverse pericardial sinus. Although an attempt was made to perform OPCAB at first, the hemodynamic condition gradually deteriorated with heart disposition and transesophageal echocardiography revealed moderate to severe mitral regurgitation. We decided to perform on-pump beating CABG. A cardiopulmonary bypass was initiated and the anastomosis between RITA and OM was performed uneventfully.

**Fig. 1:**
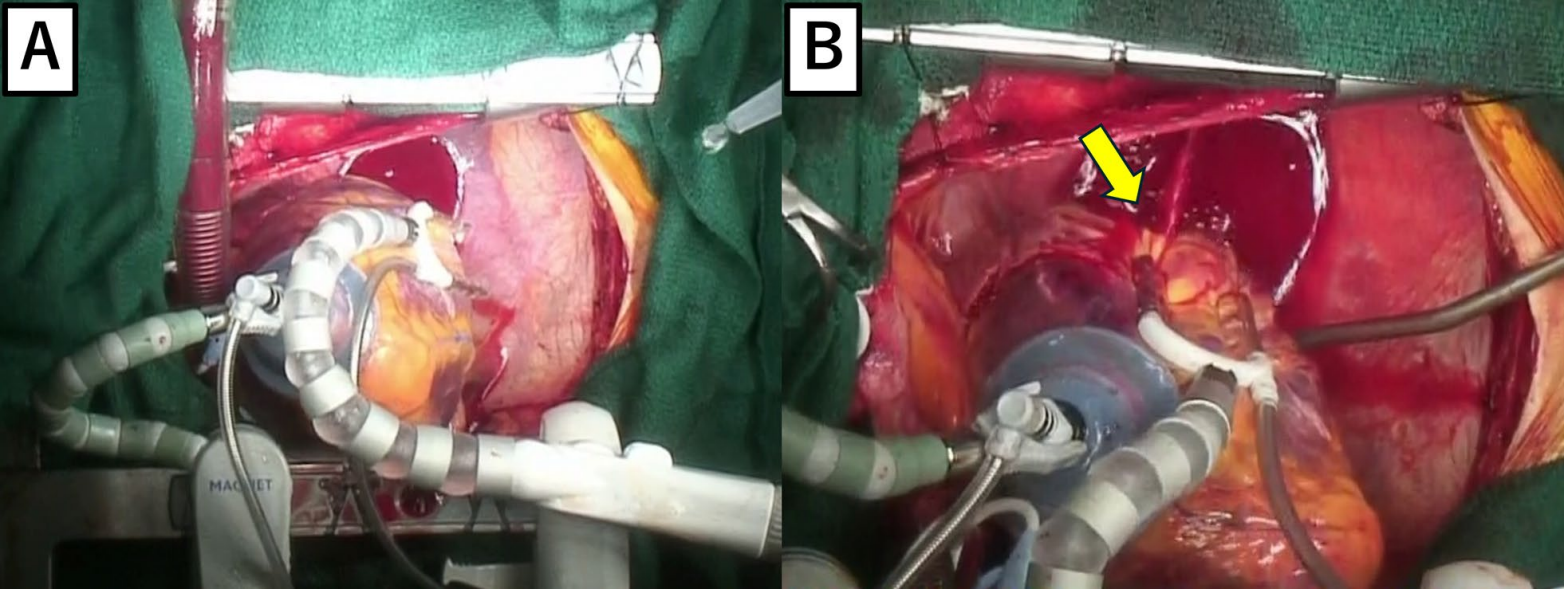
Intraoperative findings from outside the heart. **A** Just before atrial rupture, the heart was displaced cranially using a vacuum device. **B** There was a 2-cm laceration on the posterior wall of the left atrium and bleeding (arrow)

**Fig. 2:**
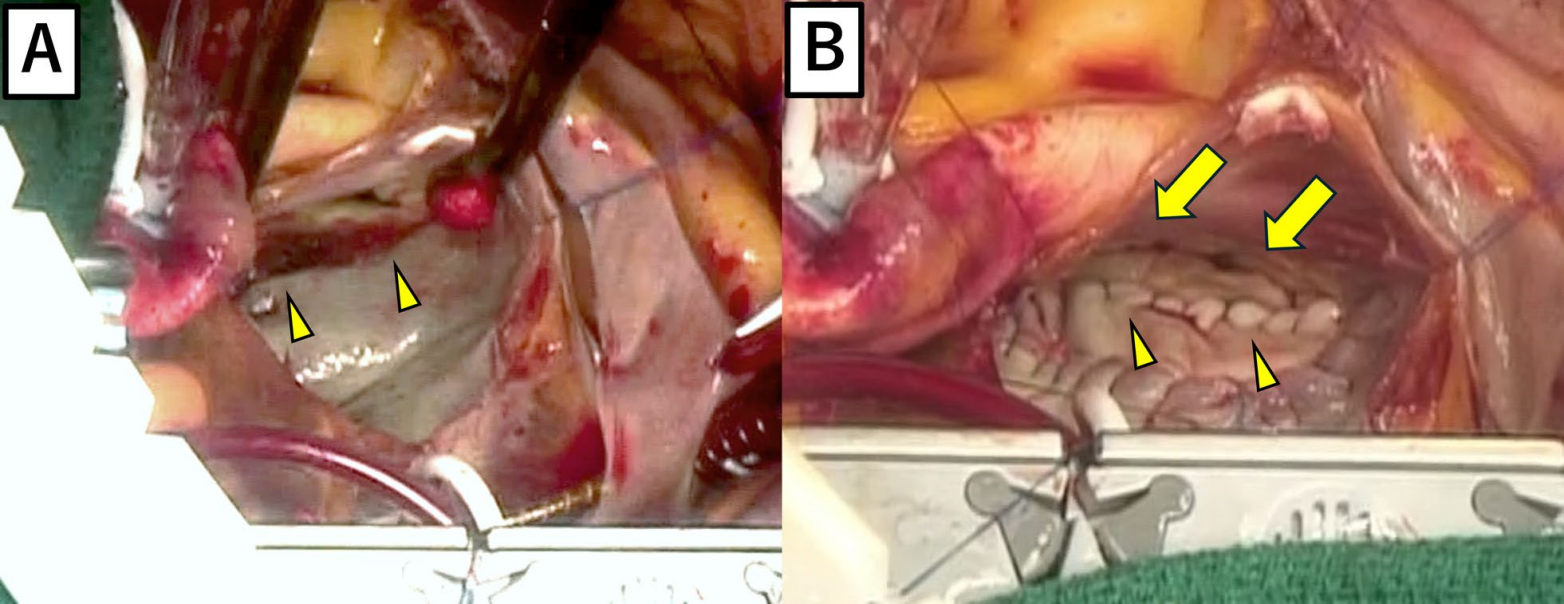
Intraoperative findings in the left atrium before and after repair. **A** Inner side of the left atrium. A 5-cm tear along the mitral annulus (arrowhead). **B** The rupture site was repaired using a bovine pericardial patch. The suture line (arrowhead) was close to the mitral annulus (arrow)

Next, to expose the right coronary artery, the positioner was attached to the apex of the heart, and the heart was transposed to the caudal side (Fig. 1A). When anastomosis between the posterior lateral ventricular branch (PLV) and SVG was about to start, sudden massive bleeding occurred in the pericardial cavity. A 2-cm-long laceration was found on the posterior wall of the left atrium between the atrioventricular groove and the left inferior pulmonary vein (Fig. 1B). A left ventricular vent was inserted and the amount of hemorrhaging was decreased. The first repair was attempted from the epicardial side using felt strips but was unsuccessful. The Heart was gone under cardioplegic arrest, the left atrium was then opened through a transseptal approach. A 5-cm fissure was found beside the posterior mitral annulus (Fig. 2A) and repaired with an oval bovine pericardial patch using 4-0 polypropylene continuous sutures (Fig. 2B). Despite intact mitral valve leaflets, substantial regurgitation was observed on the water test, necessitating annuloplasty with the tailor flexible annuloplasty ring (Abbott, Illinois, U.S.). The left and right atrium were closed, and then the remaining CABG was completed under cardiac arrest. Finally left internal thoracic artery to left anterior descending artery, RITA to OM, SVG to Diagonal branch, and SVG to the posterior descending artery and PLV were performed. SVGs were anastomosed to the ascending aorta. Since the RITA was interfering with the hemostasis of the left atrium, it was divided at the root and formed into a V-composite with the SVG anastomosed to a diagonal branch. Cardiopulmonary bypass was weaned off with intra-aortic balloon pumping assistance. The operation, extracorporeal circulation, and aortic clamping times were 474, 240, and 142 min, respectively.

Postoperatively, continuous hemodialysis was temporarily administered. However, he was transferred to the ward after 10 days. Computed tomography showed patency of all grafts and no pseudoaneurysm in the left atrium. An echocardiogram performed after 3 months showed that the left ventricular ejection fraction was 34% and mitral regurgitation was trivial.

## Discussion

On-pump beating CABG is often used when off-pump procedures are difficult due to hemodynamic compromise. This case describes a rare complication, left atrial rupture during on-pump beating CABG. Reports of left atrial injury associated with CABG are rare but there have been reports of atrioventricular groove laceration [1] and left atrial dissection [2]. The AV groove injury happened when the heart was rotated cranially and to the right side during the exposure of the left circumflex artery region on OPCAB. The mechanism is believed to be related to the deformation of the mitral annulus and increased left atrial pressure due to the rotation [1]. On the other hand, while left atrial dissection is typically a complication associated with mitral valve surgery [3], there are reports of left atrial dissection occurrence during CABG [3]. This occurred during a conventional CABG under cardiac arrest, and there is a suggested association with coronary sinus injury related to the insertion and use of retrograde coronary perfusion [2]. These reports may also suggest that the AV groove is a transition zone from the left ventricle to the left atrium, and is susceptible to the effects of physical stress.

In this case, we thought that the cause may have been the tension on the left atrium due to the displacement of the heart and the aggravation of mitral regurgitation, which put stress on the left atrial wall. Cardiac displacement for anastomosis distorts the mitral annulus, worsens mitral regurgitation, and increases left atrial pressure, especially during access to the posterior region of the heart [4]; other studies have reported that cardiac displacement increases pulmonary arterial pressure and causes left atrial enlargement [5, 6]. The atrial rupture in this case probably occurred due to the worsened mitral regurgitation jets affecting the left atrial wall under the stress of displacement. It is considered that tension may be applied to the myocardium between the apical positioner and the pulmonary veins, potentially causing injury to the AV groove or posterior wall of the left atrium. In cases where the heart is enlarged and heavy, there is a risk of applying excessive traction more than necessary, careful handling during the procedure is required. Also, in patients with reduced ventricular function and mild-to-moderate mitral regurgitation, the risk of mitral regurgitation worsening during heart dislocation must be considered. Intraventricular decompression with left ventricular ventilation may reduce this risk, especially during on-pump beating CABG.

Managing left atrial rupture is challenging because the tear size is possibly larger than it appears from the outside. In addition, patch repair from the intracardiac side is necessary for reliable hemostasis, and cardiac arrest is required. In our case, we avoided direct closure due to tissue fragility and used the pericardial patch for repair. In addition, concomitant mitral annuloplasty may be considered to manage potential deformities resulting from left atrial repair when the site of rupture is close to the mitral valve.

## Conclusions

Left atrial rupture during on-pump beating CABG is a rare complication. Intracardiac decompression with left ventricular venting is useful in patients with low left ventricular function and mitral regurgitation. Repair from the inside of the left atrial lumen should preferably be performed during cardiac arrest. Mitral valvuloplasty should be considered in such cases.

## Data Availability

The data supporting this study's findings are available from the corresponding author upon reasonable request.

## Abbreviations

CABG: Coronary artery bypass grafting
OPCAB: Off-pump coronary artery bypass grafting
SVG: Saphenous vein graft
OM: Obtuse marginal branch
RITA: Right internal thoracic artery
PLV: Posterior lateral ventricular branch
